# The widespread role of non-enzymatic reactions in cellular metabolism

**DOI:** 10.1016/j.copbio.2014.12.020

**Published:** 2015-08

**Authors:** Markus A Keller, Gabriel Piedrafita, Markus Ralser

**Affiliations:** 1 Department of Biochemistry and Cambridge Systems Biology Centre, University of Cambridge, 80 Tennis Court Road, CB2 1GA, Cambridge, UK; 2 MRC National Institute for Medical Research, The Ridgeway, Mill Hill, NW7 1AA, London, UK

## Abstract

Enzymes shape cellular metabolism, are regulated, fast, and for most cases specific. Enzymes do not however prevent the parallel occurrence of non-enzymatic reactions. Non-enzymatic reactions were important for the evolution of metabolic pathways, but are retained as part of the modern metabolic network. They divide into unspecific chemical reactivity and specific reactions that occur either exclusively non-enzymatically as part of the metabolic network, or in parallel to existing enzyme functions. Non-enzymatic reactions resemble catalytic mechanisms as found in all major enzyme classes and occur spontaneously, small molecule (e.g. metal-) catalyzed or light-induced. The frequent occurrence of non-enzymatic reactions impacts on stability and metabolic network structure, and has thus to be considered in the context of metabolic disease, network modeling, biotechnology and drug design.

## Highlights

• Non-enzymatic reactions are widespread and integral part of metabolism.
• Non-enzymatic metabolic reactions occur either spontaneously or small molecule catalyzed.
• They subdivide between broad/unspecific, and specific reactions that contribute to metabolism.
• Specific reactions occur both, exclusively non-enzymatically or parallel to enzymes.
• Non-enzymatic reactions affect drug design and network reconstruction.

## Graphical abstract

**Figure:**
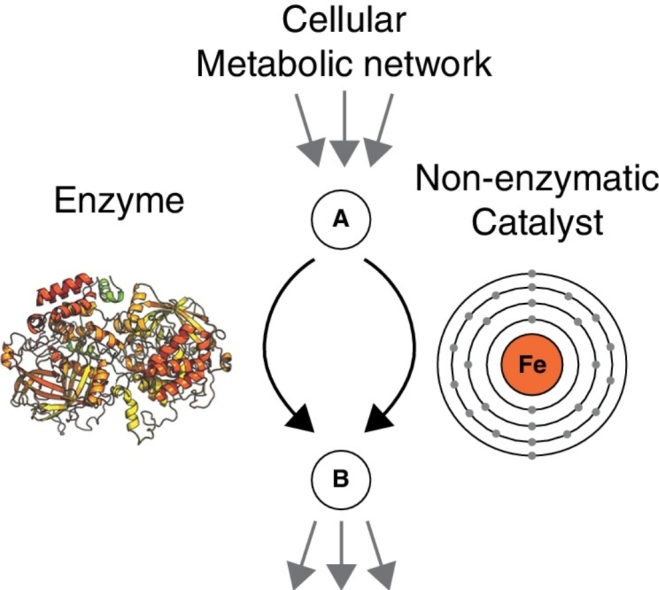

**Current Opinion in Biotechnology** 2015, **34**:153–161

This review comes from a themed issue on **Systems biology**

Edited by **Sarah Maria Fendt** and **Costas D Maranas**

For a complete overview see the Issue and the Editorial

Available online 22nd January 2015

**http://dx.doi.org/10.1016/j.copbio.2014.12.020**

0958-1669/© 2015 The Authors. Published by Elsevier Ltd. This is an open access article under the CC BY license (http://creativecommons.org/licenses/by/4.0/).

## Introduction

The metabolic network originates from a low number (or one) of ancestral forms, and all living organisms share core reaction sequences and structural properties in their metabolic networks [1]. Glycolysis and gluconeogenesis, pentose phosphate pathway (PPP) and tricarboxylic acid (TCA) cycle are central metabolic pathways and exemplary for the conservation of metabolism [2, 3]. Their products glucose, pyruvate, ribose-5-phosphate and erythrose-4-phosphate are common precursors for amino acids, lipids and nucleotides. Despite the high level of conservation of the reactions, the participating enzymes have however multiple origins. Sequences of glycolytic enzymes differ between Archaea and Bacteria/Eukaryotes [2, 4••, 5]. This divergence can be explained by both, independent evolutionary origins of enzymes and stepwise replacement of ancestral enzymes by modern forms [5]. Both scenarios require an initial reaction sequence as ‘template’, as evolution can only select for a functional product (‘end-product problem’ [6]). The initial metabolic pathway can thus either evolve backward from the advantageous end-product (retro-evolution), provided that precursors are formed non-enzymatically [6], or by improving a non-enzymatic reaction sequence starting from its most rate-limiting step [7]. A plausible primordial base can be traced for glycolysis and the PPP, as several of their reactions can be replicated with metal catalysts, in particular Fe(II), under conditions reproducing the ocean chemistry of the Archean world [8]. Fe(II) was broadly available before oxygenation of the early Earth [9], implying a scenario for the first glycolytic enzymes being simple iron-binding RNA or oligopeptide molecules, which would have possessed the potential of enhancing many reactions now found in central metabolism [7, 8] (Box 1).

## Three classes of non-enzymatic reactions contribute to modern cellular metabolism

**Figure 1:**
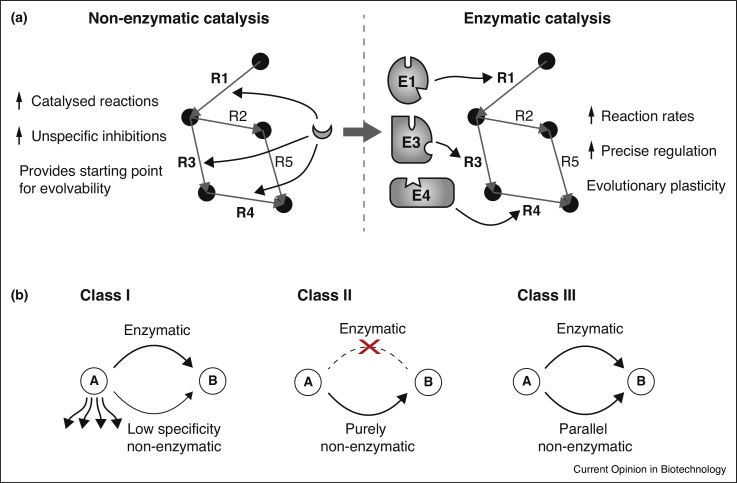
Non-enzymatic reactions in cellular metabolism. **(a)***Evolution*. Non-enzymatic reactions (R1, R3, R4, left panel) provide a template for the evolutionary selection of enzymes (E1, E3, E4). Enzymes can achieve higher substrate specificity and reaction rate, can be regulated and decrease the dependency on rare catalysts. **(b)***Three classes of non-enzymatic reactions dominate in modern metabolism*. The presence of enzymes does not prevent or replace non-enzymatic metabolic reactions, which divide into three classes: Class I reactions are non-specific and act on a broad range of substrates, Class II reactions are specific and occur exclusively non-enzymatic as part of the metabolic network, while Class III reactions occur simultaneously in an enzymatic and non-enzymatic manner.

It is important to emphasize that the same thermodynamic principles apply for non-enzymatic and enzymatic reactions, and every enzymatic reaction can occur in principle also non-enzymatically [10^•^]. Non-enzymatic reactivity of metabolites is a well-known phenomenon since the beginning of enzymology (Figure 1a). However, network topologies or genome-scale metabolic models were not prevailing research questions until the event of Systems Biology, and until recently for many cases the metabolic role of non-enzymatic reactions obtained little attention.

The presence of enzymes does not prevent non-enzymatic reactions to occur across the metabolic landscape. On the basis of their principal mode of action, we divide non-enzymatic reactions into three classes (Figure 1b): Class I reactions present broad chemical reactivity and low specificity. These include Maillard-reactions, a conjugation of amino group-containing compounds (e.g. amino acids) and sugars [11], oxidation reactions driven by reactive oxygen species (ROS) and non-enzymatic covalent modifications of lipids and proteins (alkylation, glycosylation and acetylation) [12]. These indiscriminate reactions are not the main focus of this review; nonetheless, they have a strong effect on cellular physiology and are important driving forces for evolution.

Other non-enzymatic reactions are highly specific and are integral part of the metabolic network. Class II reactions occur purely non-enzymatically. A well-known example is the maturation of vitamin D_3_ where a precursor is transported to the skin, to be converted by UV light to previtamin D_3_ [13^•^]. Most Class II reactions are spontaneous reactions which do not depend on a catalyst or an atypical energy source, such as the next downstream reaction in vitamin D_3_ biosynthesis, in which previtamin D_3_ undergoes spontaneous isomerization forming vitamin D_3_ [13^•^].

**Figure 2:**
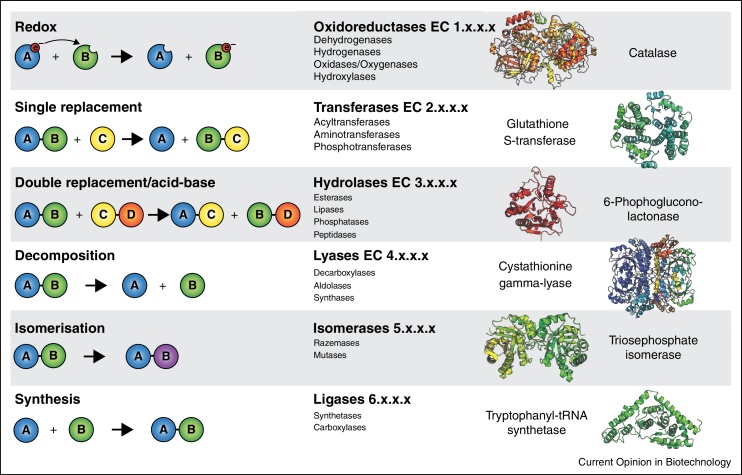
Class III non-enzymatic reactions occur in parallel to the six main classes of enzymes. Non-enzymatic counterparts of six enzymes representatives to the six general chemical reaction types/main classes of enzymatic reactions (top hierarchical level of enzyme commission number). Enzymes are exemplified by crystallographic structures illustrated in pymol; Protein Structure Databank (PDB, accession codes: 8CAT (catalase [19]), 1PKW (human glutathione transferase A1-1 [66]), 2J0E (6-phosphogluconolactonase [67]), 1N8P (PLP-dependent cystathionine gamma-lyase [68]), 4OWG (triosephosphate isomerase [53]) and 2G36 (iron–sulfur cluster containing tryptophanyl-tRNA synthetase [39]).

**Figure 3:**
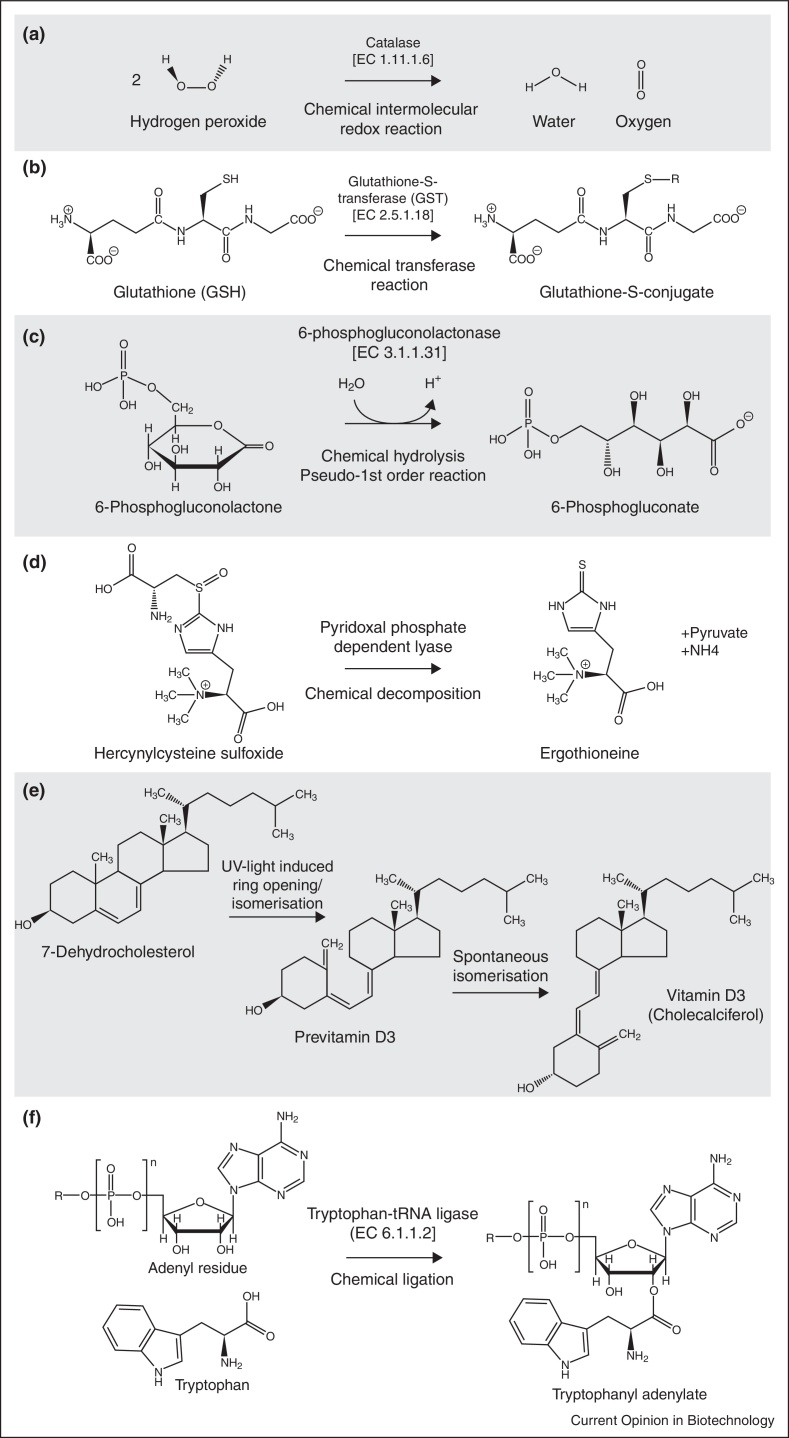
Examples for biologically important non-enzymatic chemical reactions. **(a)** The intermolecular redox reaction of two hydrogen peroxide molecules into water and oxygen is catalyzed non-enzymatically or by catalase (EC 1.11.1.6). **(b)** The thiol group of GSH is an acceptor of electrophiles allowing the formation of glutathione-S-conjugates, either non-enzymatically or through GSTs (EC 2.5.1.18). **(c)** 6-phosphogluconolactone is hydrolyzed to 6-phosphogluconate in the PPP spontaneously, or through 6-phosphogluconolactonase (EC 3.1.1.31), whose main function is to prevent unwanted side-reactions. **(d)** The final step in ergothioneine biosynthesis is the decomposition of hercynylcysteine sulfoxide either by a pyridoxal phosphate-dependent lyase, or non-enzymatically by pyridoxal phosphate alone. **(e)** The two final steps in cholecalciferol (vitamin D_3_) biosynthesis are non-enzymatic isomerizations, one UV-light dependent and one spontaneous. For both reactions no enzymatic counterpart is known (Class II reactions). **(f)** Tryptophan-tRNA ligase or a non-enzymatic reaction conjugate tryptophan to its tRNA.

Class III non-enzymatic reactions occur parallel to enzyme functions. Class III reactions are widespread in metabolism, and indicate that many metabolic pathways descend from promiscuous or non-enzymatic precursors. Frequently, the parallel enzyme operates to prevent unwanted secondary products that would be generated in the non-enzymatic reaction (negative catalysis). Examples include the spontaneous transamination of glyoxylic acid and amino acids [14], the isomerization of propyl residues [15], the decarboxylation of aminomalonic acid [16] and the formation of oxysterols from cholesterol [17]. Class III non-enzymatic reactions occur analogous to all six major enzymatic classes, illustrated for the following examples:

(i) *Oxidoreductases*: catalase (Figure 2, Figure 3). Hydrogen peroxide (H_2_O_2_) is formed as a (by-)product in various redox reactions. In combination with Fenton chemistry, H_2_O_2_ can react into superoxide and damage proteins, RNA, DNA and lipids [18]. Catalase degrades H_2_O_2_ into water and oxygen [19]. A similar reaction is also catalyzed by metals, inorganic salts and organic compounds [20, 21]. Despite catalase is faster than the non-enzymatic reactions, the non-enzymatic catalysts are present at much higher cellular concentrations (iron: μM–mM range versus catalase that is present in the nanomolar range (i.e. 38 nM) ; [22, 23]), and thus relevant for total reactivity.
(ii) *Transferases*: glutathione-S-transferases (GST) (Figure 2, Figure 3). GSTs comprise a large family of enzymes that transfer substrates to glutathione (GSH) for cellular detoxification. Several substrates (i.e. 4-hydroxynonenal, isothiocyanates or catecholamines) conjugate to GST also non-enzymatically [24, 25, 26]. The reactive GSH thiol-group provides the redox potential for both the enzymatic and non-enzymatic conjugation [26].
(iii) *Hydrolases*: 6-Phosphogluconolactonase (6PGL) (Figure 2, Figure 3). 6PGL is an enzyme of the oxidative PPP that provides NADPH and pentose metabolites [27]. Spontaneous hydrolysis of 6-phosphogluconolactone into 6-phosphogluconate occurs at rates that would allow a considerable PPP flux [28]. The presence of 6PGL additionally accelerates flux, but predominantly prevents the formation of undesired side-products produced when 6-phosphogluconolactone reacts with amino acids, lipids, polyamines or alcohols [28]. Also other lactonases such as aldonolactonase (l-ascorbate biosynthesis) [29] and hydrolase-type reactions such as acyl phosphatases [28], epoxide hydrolases [30] and lipoxygenase (colneleic acid degradation) [31] possess analogous non-enzymatic reactions.
(iv) *Lyases*: ergothioneine and tetrahydrobiopterin (BH4) (Figure 2, Figure 3). Ergothioneine is a metabolite produced by bacteria and fungi, and actively taken up by mammalian cells [32]. Its biosynthesis involves a threefold methylation of the alpha-amino group of histidine, followed by cysteinylation of the histidine side chain [33]. The final reaction is catalyzed by a pyridoxal phosphate-dependent lyase, or occurs in a non-enzymatic manner ([33], supporting information). Another example for a non-enzymatic lyase reaction is found in the BH4 salvage pathway that regenerates BH4 with the help of dihydropterin-4a-carbinolamine dehydratase and dihydropteridine reductase (DHPR). When DHPR activity becomes limiting, the intermediate 6,7-dihydrobiopterin rearranges non-enzymatically allowing salvage of BH4 [34].
(v) *Isomerases*: glycolysis, PPP and isomerization in vitamin D biosynthesis (Figure 2, Figure 3). Non-enzymatic isomerization reactions are frequently observed, for instance between glycolytic intermediates fructose-6-phosphate, glucose-6-phosphate or mannose-6-phosphate [35] as well as PPP metabolites ribulose-5-phosphate, xylulose-5-phosphate and ribose-5-phosphate [8]. Another example is the isomerization of glyceraldehyde-3-phosphate and dihydroxyacetone phosphate. Here, the enzyme triosephosphate isomerase (TPI) speeds up the reaction and prevents the formation of toxic methylglyoxal [36]. In some instances UV light provides the activation energy. Bacteria use UV-induced trans/cis isomerizations in Class I rhodopsins as a source of energy [37], mammalian cells exploit photo-induced trans/cis isomerizations in the eye pigment retinal [38]. As previously mentioned, non-enzymatic isomerizations are also required in the maturation of vitamin D [13^•^].
(vi) *Ligases*: tRNA loading and hydrolysis (Figure 2, Figure 3). The process of tRNA-amino acid ligation, tRNA loading, is enzymatically attributed to aminoacyl tRNA synthetases and crucial for ribosome function and protein biosynthesis [39]. tRNAs also ligate (and hydrolyse) non-enzymatically, a reaction best studied for tryptophan-tRNA ligase [40, 41]. Of note, non-enzymatic ligation reactions have lower substrate specificity and can cause tRNA mischarging [42].

## The success of enzymes in replacing non-enzymatic reactions

**Figure 4:**
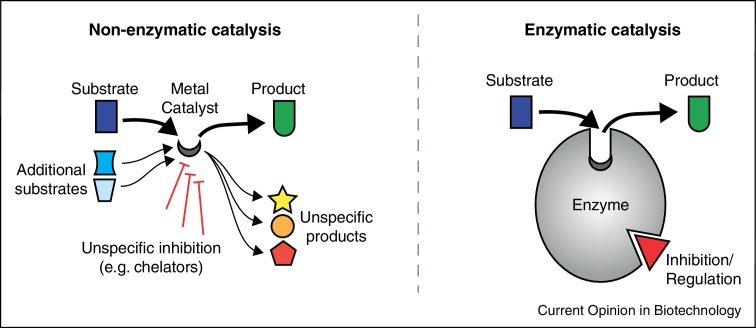
The success of enzymatic catalysis in cellular metabolism. The conversion of a substrate into its product can be mediated by non-enzymatic catalysts (organic/metal, left panel) or enzymatically (right panel). Non-enzymatic catalysts accelerate reactions but often display a broad substrate and product spectrum. While this variability is exploited during evolution, metabolism gains efficiency with enzymes that are specific and are less dependent on availability of rare catalysts. Moreover enzymes allow to more precisely regulate certain reactions and therewith to distribute flux through-out the metabolic network.

Small-molecule compounds are excellent catalysts in order to accelerate chemical reactions. However, in biological systems other important constraints apply in parallel, and explain the success of enzymes (Figure 4):

(i) *Limited catalyst availability*: metal ions and minerals may qualify as excellent catalysts, but often are not readily available. Molybdenum for instance is low concentrated in most ecosystems, while Fe(II) became limited during earth history [9]. Enzymatic catalysts render metabolism less dependent on the presence of rare and/or insoluble molecules [8].
(ii) *Substrate specificity*: enzyme folds can achieve substantial substrate specificity and differentiate similar molecules, for instance glucose-6-phosphate from fructose-6-phosphate [43]. This allows specificity and chemical compartmentalization: analogous reactions can occur in parallel when relying on structurally distinguishable co-factors such as NADP(H) and NAD(H) [44].
(iii) *Negative catalysis*: enzymes can function to prevent side reactions, a concept termed negative catalysis [45]. Negative catalysts are essential for the efficiency of metabolism and of particular medical importance, as mutations within these enzymes are causative for a number of inherited metabolic diseases. Examples include mutations of isocitrate dehydrogenase (IDH) that lead to an increased release of the side product d-2-hydroxyglutarate in cancer (IDH1 and IDH2), or in d-2-hydroglutaric aciduria (IDH2) [46, 47•], and TPI where pathogenic mutations have been associated with the production of methylglyoxal [36].
(iv) *Regulation of metabolism*: surviving starvation and stress situations as well as development of multicellular organisms requires regulation of metabolism. Non-enzymatic catalysts are however not readily tunable. Enzymes instead allow multiple levels of control, including allosteric regulation, cooperativity, post-translational modifications and genetic/transcriptional regulation [48, 49••].

## When enzymatic catalysts have their limits

The dominance of enzymes impressively demonstrates their benefits for living organisms. Nonetheless, also enzymatic catalysis creates limitations. First, enzymatic structures can be highly temperature sensitive, a reason for the low thermo-tolerance of most species. Secondly, enzymatic catalysis is costly, as protein biosynthesis is one of the most energy-consuming cellular processes [50]. Third, enzymes are prone to chemical modifications, and need constant replacement, a cause of ageing and cancer [51]. Less intuitively, a forth constraint arises from their high specificity: structurally similar molecules can bind to catalytic pockets without being metabolized; turning harmless molecules into metabolic inhibitors. An example for the latter is TPI. *In vitro* regarded as a perfect enzyme solely limited by diffusion rate [52], it is *in vivo* competitively inhibited by molecules with high structural similarity to its substrates, as for example phosphoenol pyruvate (PEP) [53]. Some enzymes evolved structural features to limit such problems, for instance fatty aldehyde dehydrogenase possesses a gatekeeper helix that prevents nonspecific metabolites from diffusing into its catalytic center [54].

## Non-enzymatic reactions as a challenge for genome-scale metabolic modeling

Metabolic modeling has become popular in biotechnology, for instance helping to understand function and behavior of metabolic systems in constraint-based flux balance analyses (FBA) [55^•^]. Limited experimental evidence is a restraining factor when constructing models for most species. Metabolic networks are therefore assembled on the basis of sequence homology [56]. A few changed residues can however alter substrate specificity of enzymes [57^••^], and metabolic reconstruction using comparative genomics further fails to capture non-enzymatic reactions [58]. The global problem is less obvious in frequently studied model organisms, since for many non-enzymatic reactions an enzymatic counterpart exists (Class III). However, with increasing phylogenetic distance the predictive power of comparative genomics decreases. This problem appears most relevant in thermophilic microorganisms [2], as it is an intrinsic property of non-enzymatic reactions to occur faster with increasing temperatures. Thus, in a Class III reaction the enzymatic contribution to the total rate becomes less important with temperature. For instance, non-enzymatic, metal-catalyzed PPP reactions strongly accelerate at temperatures above 60 °C [8]. The absence of a PPP enzyme in a thermophile is thus not necessarily an indicator of whether this pathway is present or not.

Also unspecific Class I reactions have a strong influence on the metabolic network, especially when cells are exposed to increased temperature and stress conditions. Exploring their system wide impact is — due to their unspecific nature and the formation of various non-enzymatic metabolic products — a difficult task. Class I reactions require sophisticated metabolite repair strategies [59], that can represent evolutionary branching points for novel metabolic pathways.

In this respect, it is worth mentioning the possible non-enzymatic contribution to metabolic transport processes. Non-enzymatic transport is often referred to as ‘membrane leakage’, a term which however falls short of taking into account that specific physical properties of metabolite and membrane composition determine the probability of a molecule to membrane diffuse. Metabolites can be attracted to specific physico-chemical environments resulting in their accumulation, causing spontaneous spatial segregation, hydrophilic–lipophilic phase separation, and membrane adsorption [60]. A well understood case is the phase separation of polar and apolar metabolites, a process of potential relevance for intracellular metabolism. Another aspect is the possible free diffusion across membranes, which is often triggered by external factors such as heat, pH or oxidative stressors [61]. Indeed, the evolution towards the modern, tightly hydrophobic lipid composition of membranes may have only been facilitated upon the appearance of membrane protein channels, transporters and pumps [62]. Membranes were likely more permeable in early organisms, which thus could more likely exploit non-enzymatic chemistry in membrane transport and separation processes [63].

## Non-enzymatic metabolic reactions and drug design

Metabolism offers a plethora of targets for developing the next generation of pharmaceuticals to treat cancer and neurodegenerative disorders [64, 65]. Nonetheless, no ‘cancer-specific’ or ‘Alzheimer-specific’ metabolic network exists, therefore respective therapeutics need to operate in narrow, intelligently designed therapeutic windows. The presence of non-enzymatic reactions puts an additional constraint on the selection of drug targets. Class I/II non-enzymatic reactions are largely not targetable, as they are a consequence of the chemical properties of the metabolites. Interesting cases are the Class III reactions, as the non-enzymatic reaction is not affected by targeting of the parallel enzyme. At best, this would simply limit the efficiency of the drug. Many Class III enzymes are however negative catalysts. Their inhibition increases the production of unwanted by-products. This can cause problematic side effects, but may create a possibility to overflow cells with toxic metabolites to target for example cancer cells or bacterial pathogens.

## Concluding remarks

Non-enzymatic reactions occur frequently within the metabolic network. We distinguish reactions with low substrate specificity (Class I) from specific reactions that occur exclusively non-enzymatically (Class II), and reactions that occur analogous to the six principal enzyme categories (Class III). Providing a template for the evolutionary selection of metabolic enzymes, non-enzymatic reactions did form the basis for the evolution of metabolism. They have however never been depleted from the chemical environment of the cell, and occur within or in parallel to exiting enzyme function. Non-enzymatic reactions should thus not be regarded as cumbersome side effects, but as integral part of the metabolic network. As such, they play a role in human metabolic disease, and have to be considered in genome-scale reconstructions of metabolic networks, in particular in extremophiles, when designing biotechnological models, and further, when selecting enzymes for drug targeting and considering the side-effects of drugs. Non-enzymatic reactions are thus central for understanding fundamental problems in biology and play an essential role in cellular metabolism, human health, and ageing.

## References and recommended reading

Papers of particular interest, published within the period of review, have been highlighted as:

• of special interest
•• of outstanding interest

## Box 1

Evolutionary replacement of metal catalysis with protein catalysts: the case of ribulose 5-phosphate 3-epimerase, which exists as an Fe(II)-dependent form and an iron-independent form. While in many bacteria the Fe(II)-dependent form dominates, in higher eukaryotes it is the iron independent form [69]. As the isomerization of ribulose 5-phosphate is catalyzed by Fe(II) also in the absence of the enzyme [8] indicates that the iron-dependent form is the older enzyme. Exchanging the Fe(II)-dependent with the metal-independent form of the epimerase does provide selective advantage: the Fe(II) catalyst in the bacterial epimerase gets readily oxidized, in fact this enzyme is the first one damaged when *Escherichia coli* is exposed to oxidants; the Fe(II) dependence of RPE is thus one major cause why *E. coli* cells can tolerate only moderate H_2_O_2_ levels [70].
